# Novel Aspects of Polynucleotide Phosphorylase Function in *Streptomyces*

**DOI:** 10.3390/antibiotics7010025

**Published:** 2018-03-18

**Authors:** George H. Jones

**Affiliations:** Department of Biology, Emory University, Atlanta, GA 30322, USA; george.h.jones@emory.edu

**Keywords:** polynucleotide phosphorylase, *Streptomyces*, ribonuclease, regulation, promoter, RNA decay, polyadenylation, (p)ppGpp, antibiotic

## Abstract

Polynucleotide phosphorylase (PNPase) is a 3′–5′-exoribnuclease that is found in most bacteria and in some eukaryotic organelles. The enzyme plays a key role in RNA decay in these systems. PNPase structure and function have been studied extensively in *Escherichia* *coli*, but there are several important aspects of PNPase function in *Streptomyces* that differ from what is observed in *E. coli* and other bacterial genera. This review highlights several of those differences: (1) the organization and expression of the PNPase gene in *Streptomyces*; (2) the possible function of PNPase as an RNA 3′-polyribonucleotide polymerase in *Streptomyces*; (3) the function of PNPase as both an exoribonuclease and as an RNA 3′-polyribonucleotide polymerase in *Streptomyces*; (4) the function of (p)ppGpp as a PNPase effector in *Streptomyces*. The review concludes with a consideration of a number of unanswered questions regarding the function of *Streptomyces* PNPase, which can be examined experimentally.

## 1. Introduction

Polynucleotide phosphorylase (PNPase, EC 2.7.7.8) was the first enzyme shown to synthesize polyribonucleotides [1], and for some time, it was thought to be the bacterial RNA polymerase. The enzyme was subsequently characterized in *Escherichia coli* and other bacteria, and was shown to catalyze the following reaction:

(p^5′^N^3′^OH)_X_ + Pi ⇆ (p^5′^N^3′^OH)_X−1_ + pp^5′^N

where N is any of the four bases found in RNA [2,3]. As written, the reaction depicts the phosphorolytic degradation of RNA chains, and this activity appears to reflect the major function of PNPase in vivo. The reaction is reversible, however, and PNPase will synthesize polyribonucleotide chains, using nucleoside diphosphates (NDPs), rather than triphosphates, as substrates. The polymerizing activity of PNPase played an important role in the synthesis of polyribonucleotides used to unravel the genetic code [4,5].

PNPase is found in all bacteria examined to date, except *Mycoplasma*, and is also present in eukaryotic organelles [6]. The enzyme has not been identified in Archaea [7]. In *E. coli*, PNPase and RNase II are the major 3′-exonucleases involved in RNA degradation [8]. In addition to its degradative function, PNPase plays a role in the bacterial response to environmental stresses, such as cold shock [9,10,11], is involved in biofilm formation [12,13] and virulence determination [14,15], and the activity of the enzyme is modulated by a number of small molecule effectors, at least in vitro [16,17,18,19]. 

*Streptomyces* are Gram-positive, soil-dwelling bacteria, notable for their ability to form spores and for their capacity to produce antibiotics [20,21]. Nearly 70% of all antibiotics used in clinical and veterinary medicine worldwide are synthesized as natural products by members of the genus [22]. Of particular relevance to this review, a number of biochemical and genetic features of *Streptomyces* PNPase distinguish it from its counterparts in other bacteria. In what follows, the functions of PNPase in *Streptomyces* will be explored. The reader is referred to several excellent reviews as sources of additional information on PNPase from *E. coli* and other bacteria [2,3,23,24].

## 2. Organization and Expression of the PNPase Gene in *Streptomyces*

In *E. coli*, and other organisms that have been studied, the PNPase gene, *pnp*, is a part of an operon that also includes *rpsO*, the gene for ribosomal protein S15 [9,25,26]. That operon is transcribed from two promoters in *E. coli*, designated P*rpsO* and P*pnp* [25,27]. P*rpsO* is situated upstream of the *rpsO* gene, and P*pnp* is located in the intergenic region between the two genes. Transcription from P*rpsO* ends at a rho-independent terminator and produces the *rpsO* transcript, but transcription through this terminator occurs with significant frequency and produces a readthrough transcript, containing both *rpsO* and *pnp*. Transcription from the intergenic promoter, P*pnp*, produces a transcript containing *pnp* only [25,27]. In addition to the *rpsO* terminator, the *rpsO–pnp* intergenic region contains a second stem–loop that functions as a processing site for the double strand specific endoribonuclease, RNase III. RNase III processing plays an important role in *pnp* expression [25,28,29,30].

**Figure 1:**
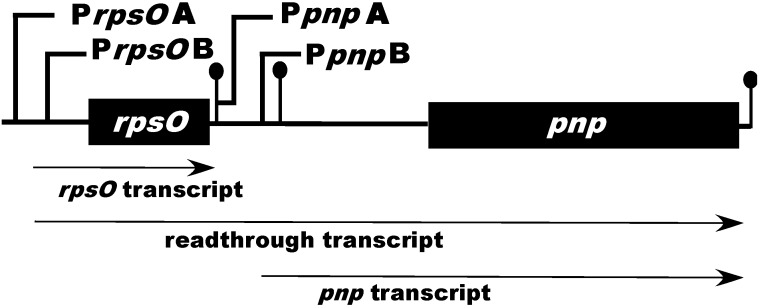
Schematic representation of the *Streptomyces coelicolor rpsO–pnp* operon. P*rpsO*A, B and P*pnp*A, B represent the upstream and intergenic promoters found in *S. coelicolor*, respectively. The ball-and-stick structures immediately following *rpsO* and *pnp* represent rho-independent transcription terminators. The ball-and-stick structure just upstream of *pnp* represents the intergenic hairpin which is cleaved by RNase III. The diagram is not drawn to scale.

Our interest in the mechanisms of RNA decay in *Streptomyces* led to an examination of the transcriptional organization of the *rpsO–pnp* operon in *Streptomyces coelicolor*, the paradigm for biological studies in the genus. To our surprise, primer extension analysis of RNAs isolated from a parental strain of *S. coelicolor* and from an RNase III null mutant revealed not two, but four extension products, suggesting the presence of four promoters within the *rpsO–pnp* operon [31]. A visual inspection of 162 putative *Streptomyces* promoters from Strohl [32] and Yamazaki et al. [33], and a group of synthetic promoters generated by Seghezzi et al. [34], indicated that all four of the 5′-ends identified by primer extension in our studies were preceded by sequences similar to the −10 and −35 regions of characterized streptomycete promoters. Promoter probe cloning of DNA fragments containing these putative promoter sequences verified that the *rpsO–pnp* operon of *S. coelicolor* is transcribed from four promoters, two situated upstream of *rpsO* (P*rpsO*A and P*rpsO*B) and two situated in the intergenic region, upstream of *pnp* (P*pnp*A and P*pnp*B, Figure 1).

**Figure 2:**
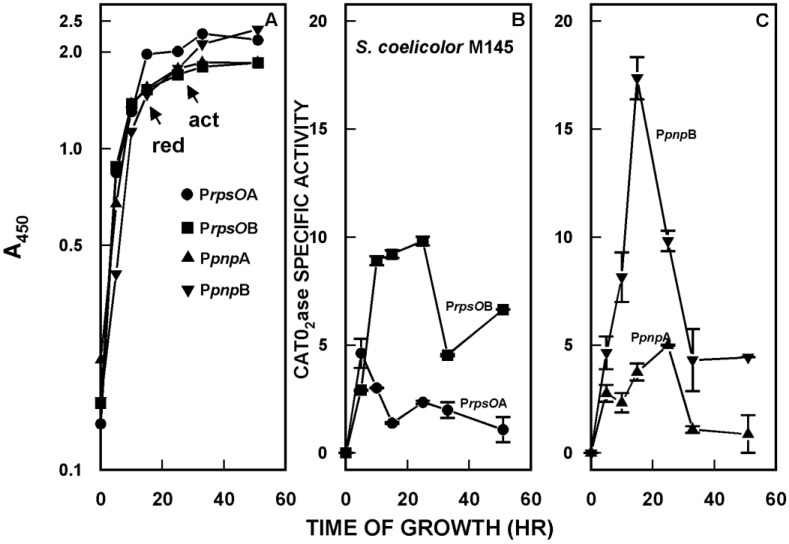
(**A**) Growth of the *S. coelicolor* strains containing promoter probe constructs. Growth was measured as the increase in optical density at 450 nm. The arrows in the figure indicate the onset of the production of two of the secondary metabolites synthesized by *S. coelicolor*, undecylprodigiosin (red) and actinorhodin (act). (**B**) Catechol dioxygenase (CATO_2_ase) activity of mycelial extracts of *S. coelicolor* derivatives containing the putative *rpsO*A and *rpsO*B promoters, cloned in the promoter probe vector pIPP2 [35]. Mycelium was harvested at the indicated times, disrupted by sonication, and following centrifugation, supernatants were assayed for catechol dioxygenase, as described previously [31,35]. The catechol dioxygenase gene is the reporter in the promoter probe vector [35]. (**C**) CATO_2_ase activities of extracts of strains containing the putative *pnp*A and *pnp*B promoters. The results shown are the averages of duplicate assays from two independent experiments ± SEM. This figure is reprinted from *Gene*, 536, Patricia Bralley, Marcha L. Gatewood, George H. Jones, Transcription of the *rpsO–pnp* operon of *Streptomyces coelicolor* involves four temporally regulated, stress responsive promoters. 177–185, Copyright (2014), with permission from Elsevier [31].

Of particular interest in this analysis was the observation that the four promoters were temporally regulated. That is, the activity of the four promoters varied with time after inoculation of liquid cultures and the variations were promoter-specific, as shown in Figure 2. In terms of maximal activity, P*pnp*B was most active followed by P*rpsO*B, P*pnp*A, and P*rpsO*A.

A major mechanism for the modulation of promoter activity in bacterial systems involves the use of alternative sigma factors by RNA polymerase. The *S. coelicolor* genome encodes over sixty alternative sigma factors, many of which play roles in differentiation and responses to stress (reviewed in [36]). It was of considerable interest to determine whether the four *rpsO–pnp* promoters might require alternative sigma factors for transcription in *S. coelicolor*.

To this end, we obtained null mutants for a number of sigma factors, including σ^B^, σ^H^, and σ^L^ [36], and their corresponding parental strains. We transferred the *rpsO–pnp* promoter probe constructs to each strain and measured promoter activity. The results obtained for the σ^H^ and σ^L^ mutants were quite similar to those for the parental strain of *S. coelicolor*, that is, the same pattern of temporal regulation was observed in both of these sigma factor mutants as in the parental strain (cf. Figure 2). In marked contrast, the P*rpsO*A, P*rpsO*B, and P*pnp*B promoters were completely inactive in the σ^B^ mutant. P*pnp*A, however, was as active in the σ^B^ mutant as in the parental strain, and showed a similar pattern of temporal expression [31]. This result indicates that P*rpsO*A and B and P*pnp*B are dependent on σ^B^ for activity, and suggests that these promoters are transcribed by an RNA polymerase holoenzyme containing σ^B^.

**Figure 3:**
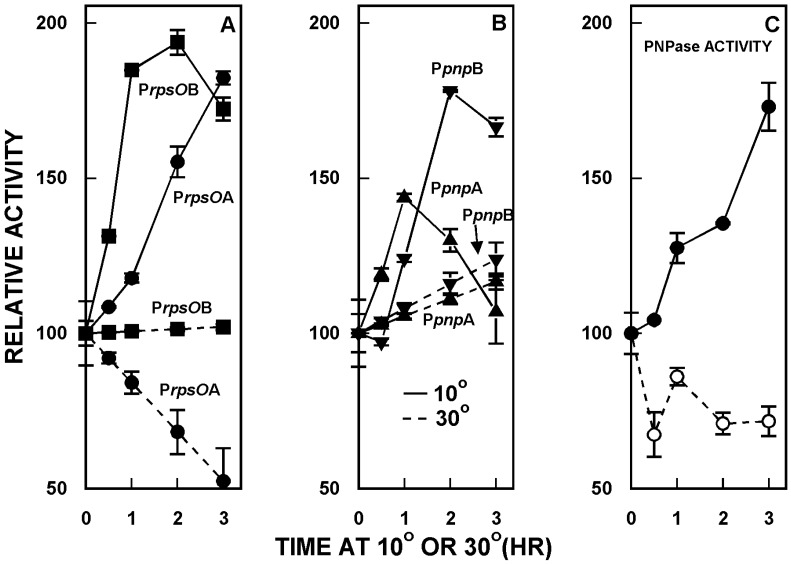
Cold shock responses of *S. coelicolor*. Derivatives containing the *rpsO–pnp* promoter probe constructs were grown and 30 °C, and half of each culture was then shifted to 10 °C. Mycelium was harvested at the indicated times, disrupted by sonication, and following centrifugation, supernatants were assayed for promoter activity, as described [31,35]. Panel **C** shows the results of PNPase polymerization assays. In Panels **A** and **B**, PNPase promoter activities are expressed relative to the activity measured at 30 °C at zero time, immediately before the shift to 10 °C. The results shown are the averages of duplicate assays from two independent experiments ± SEM. In the first experiment, PNPase levels were measured in *S. coelicolor* containing P*rpsO*A and in the second, PNPase levels were measured in the derivative containing P*pnp*B. This figure is reprinted from Gene, 536, Patricia Bralley, Marcha L. Gatewood, George H. Jones, Transcription of the *rpsO-pnp* operon of *Streptomyces coelicolor* involves four temporally regulated, stress responsive promoters. 177–185, Copyright (2014), with permission from Elsevier [31].

PNPase is a cold shock protein in many bacteria, and it has been shown that cold shock leads to an increase in PNPase levels in *E. coli* and other organisms [9,10,11]. It was of interest to determine whether PNPase levels increased in cold shock in *S. coelicolor*, and whether any such increase reflected changes in the activity of the *rpsO–pnp* promoters. As shown in Figure 3C, PNPase activity increased significantly (two-fold) in *S. coelicolor* over three hours of cold shock at 10 °C. This increase in activity was accompanied by an increase in the activities of all four of the *rpsO–pnp* promoters (Figure 3A,B) as compared with their activities at the normal growth temperature, 30 °C.

Thus, PNPase is a cold shock protein in *Streptomyces*, and the cold shock response involves changes in the activities of the promoters responsible for transcription of the *rpsO–pnp* operon [31].

## 3. PNPase Function as an RNA 3′-Polyribonucleotide Polymerase in *Streptomyces*

As is the case in eukaryotes, bacterial RNAs have oligo- and polyribonucleotide tails at their 3′-ends, and these tails are added post-transcriptionally (reviewed in [37]). In *E. coli*, the primary enzyme responsible for the synthesis of these tails is poly(A) polymerase I (PAP I), and the tails are composed primarily of A residues [38]. In bacteria, poly(A) tails function to facilitate RNA degradation as the major 3′-exoribonucleases, viz. RNase II and PNPase in *E. coli*, digest these tails processively in vitro and in vivo [8,39].

It was shown some years ago by Mohanty and Kushner that an *E. coli* mutant lacking PAP I still added tails to the 3′-ends of its RNAs. However, these tails were not composed exclusively of A residues; the tails contained G, C, and U residues as well, i.e., they were heteropolymeric [40]. Mohanty and Kushner demonstrated that the enzyme responsible for the synthesis of these heteropolymeric tails was none other than PNPase [40]. Thus, even in the absence of PAP I, PNPase can add 3′-tails to facilitate the degradation of cellular RNAs.

*Streptomyces* do not contain PAP I. Yet the 3′-ends of streptomycete RNAs do possess tails. Moreover, the tails are heteropolymeric in composition, like those synthesized by PNPase in *E. coli* in the absence of PAP I [41,42]. These observations, and the report that PNPase functions as the RNA 3′-polyribonucleotide polymerase in plant chloroplasts and in cyanobacteria [43,44], led to the hypothesis that PNPase played the same role in *Streptomyces* [45]. The straightforward way to test this hypothesis would be to create a *pnp* null mutant, e.g., in *S. coelicolor*, and to determine whether the mutant was still capable of adding 3′-tails to its RNAs. However, attempts to disrupt *pnp* in *S. coelicolor* and in the sister species, *Streptomyces antibioticus*, were only successful when a second copy of *pnp* was added to the genome. In other words, gene disruption attempts revealed that, unlike the situation in *E. coli* and in the Bacilli, *pnp* is an essential gene in *Streptomyces* [42].

A model for the function of PNPase as an RNA 3′-polyribonucleotide polymerase is presented in detail below.

## 4. Function of PNPase as Both an Exoribonuclease and as an RNA 3′-Polyribonucleotide Polymerase

PNPase activity is highly processive and the enzyme is impeded by stem–loop structures [46]. Streptomycete genomes are GC rich, so that enzymes involved in RNA decay may have evolved to degrade the RNAs derived from these genomes efficiently. A possible strategy for facilitating this degradation was suggested by the observation that PNPase appears to utilize its polymerizing activity to add 3′-tails to streptomycete RNAs [42,45]. It seemed possible that the enzyme might add such tails during phosphorolysis to create single stranded 3′-ends that would then function as the substrates for that phosphorolysis. If this were the case, it might be expected that nucleoside diphosphates, the substrates for polymerization, would stimulate phosphorolysis. To test this hypothesis, two model PNPase substrates were constructed from the sequence of the *rpsO–pnp* operon of *S. coelicolor*. Both substrates contained the *rpsO–pnp* terminator and the intergenic hairpin. Thus, both model substrates contained secondary structure that would be expected to impede phosphorolysis by PNPase. One substrate, designated 5601, also possessed a single stranded 3′-tail, 33 bases in length, while the other substrate, 5650, terminated at the base of the intergenic hairpin, and did not have a single stranded tail [47].

**Figure 4:**
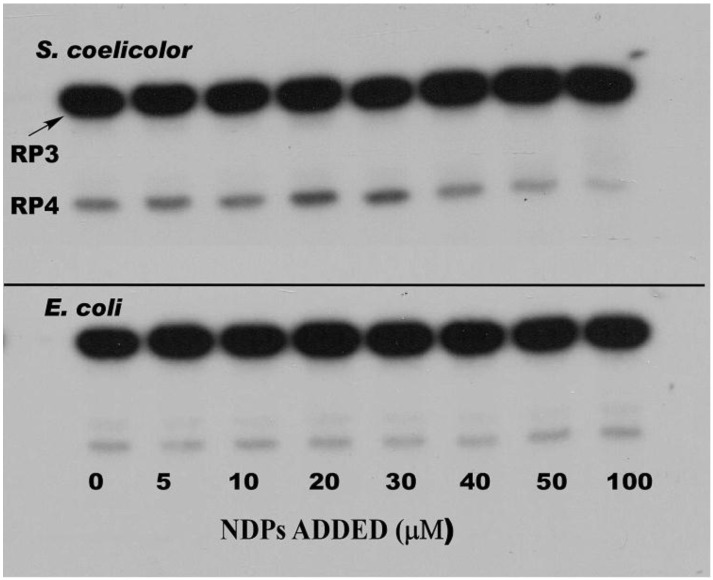
Effects of nucleoside diphosphates on the phosphorolysis of the 5650 transcript. Phosphorolysis reactions were performed as described in [47], and reaction products were separated by gel electrophoresis. The top panel shows the results obtained with *S. coelicolor* PNPase and the bottom panel results using *E. coli* PNPase. Reactions were conducted in the presence of increasing concentrations of a mixture of ADP, CDP, UDP, and GDP (nucleoside diphosphates (NDPs)) as indicated. RP3 is the 5650 transcript, and RP4 represents the product obtained by complete digestion of the intergenic hairpin in RP3 by PNPase. Note that as PNPase is highly processive [48], no intermediates with mobilities between those of RP3 and RP4 were observed. Copyright © American Society for Microbiology (*J. Bacteriol.* 190, 2008, 98–106, DOI:10.1128/JB.00327-07) [47].

The phosphorolysis of these two substrates was studied in the absence and presence of a mixture of all four nucleoside diphosphates (NDPs) with the interesting result, predicted by our hypothesis, that the NDPs, normally the substrates for the polymerizing activity of PNPase, did stimulate RNA degradation by phosphorolysis. Figure 4 shows the results of phosphorolysis of the 5650 transcript (labeled RP3 in the figure).

Analysis of the results shown in Figure 4 revealed that NDPs at 20–30 µM in phosphorolysis mixtures stimulated that reaction by 2–3 fold as compared with controls, but only when the structured RNA, 5650 (RP3) was used as the substrate [47]. NDPs had no effect on the phosphorolysis of the 5601 substrate, possessing the single stranded tail. Kinetic analyses showed that NDPs affected the *K*_m_ for phosphorolysis. Thus, the *K*_m_ value for phosphorolysis of the 5650 substrate in the absence of NDPs was 3.1 µM. This value decreased to 0.65 µM in the presence of all four NDPs at 20 µM. This latter *K*_m_ was almost identical to that obtained in the absence of NDPs for the 5601 substrate, which has a single stranded 3′-tail (0.62 µM). NDPs did not further decrease the *K*_m_ for the 5601 substrate. It is noteworthy as well, that NDPs had no effect on the phosphorolysis of either substrate by *E. coli* PNPase, and that the *E. coli* enzyme was intrinsically less active with the structured substrate than was its counterpart from *S. coelicolor* [47].

**Figure 5:**
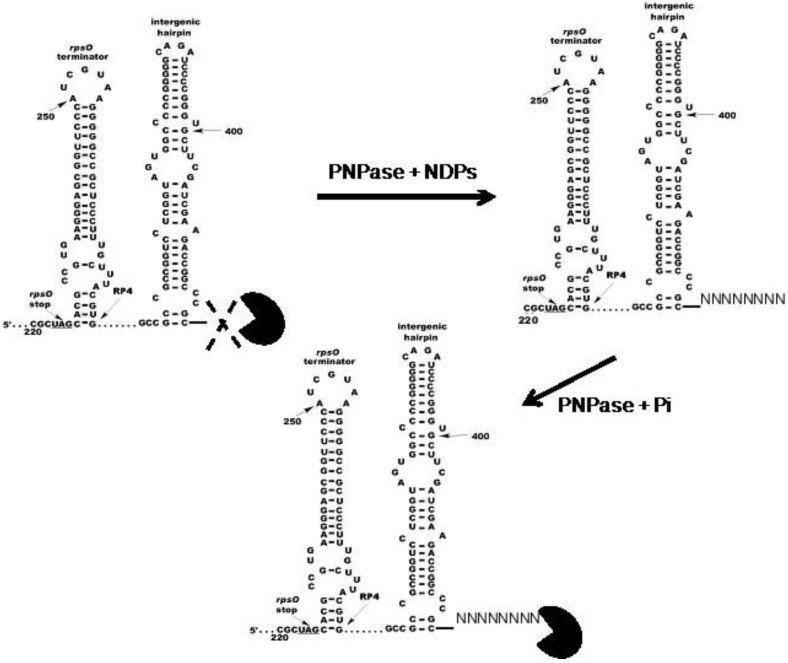
Model for the effects of NDPs on the activity *S. coelicolor* PNPase. The model posits that *S. coelicolor* PNPase (PacMan symbol) is able to phosphorolyze 5650 and other structured substrates to a limited extent in the absence of NDPs, as indicated by the dashed X. In the presence of NDPs, PNPase synthesizes unstructured 3′-tails in vivo, and these tails then provide an anchor for the enzyme, thus facilitating the digestion of structured substrates. Copyright © American Society for Microbiology (*J. Bacteriol.* 195, 2013, 5151–5159, DOI:10.1128/JB.00936-13) [49].

Our model for the explanation of the foregoing observations is shown in Figure 5 [49]. 

We posit that the stem–loops of structured substrates, like 5650, block PNPase action. The addition of short 3′-tails during phosphorolysis, or the presence of naturally occurring tails on substrates like 5601, allows for the breathing of stems and thus permits PNPase, which might otherwise stall at the stem–loops in structured substrates, to continue phosphorolysis through those structures. As indicated above, this mechanism may represent an evolutionary adaptation occasioned by the high GC content of streptomycete genomes and their transcripts.

It must be noted, however, that the evidence for the function of PNPase as a 3′-polyribonucleotide polymerase in *Streptomyces* is indirect. In vivo evidence for this function remains to be uncovered.

## 5. (p)ppGpp as a PNPase Effector

Highly phosphorylated guanine nucleotides, (p)ppGpp, guanosine pentaphosphate, and guanosine tetraphosphate, are alarmones that play a number of roles in the regulation of bacterial metabolism (reviewed in [50,51]). (p)ppGpp is synthesized by the product of the *relA* gene in *E. coli* [52], and that gene is found in *S. coelicolor* [53,54], *S. antibioticus* [55], and other streptomycetes [56]. In *Streptomyces*, ppGpp plays an important role in the regulation of antibiotic synthesis [53,54,55,56].

**Figure 6:**
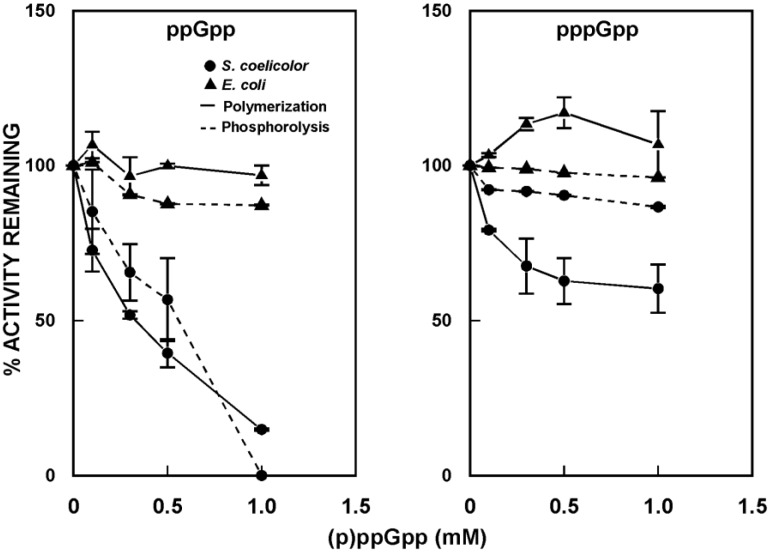
Effects of (p)ppGpp on the activity of PNPase. Polymerization and phosphorolysis reactions were performed in the absence and presence of guanosine tetraphosphate (ppGpp) or guanosine pentaphosphate (pppGpp), using purified PNPase from *S. coelicolor* and *E. coli* [59]. Results are expressed relative to the activities measured in the absence of (p)ppGpp, set arbitrarily to 100%.

PNPase from *S. antibioticus* was shown to synthesize pppGpp in vitro [57,58]. While this activity may be an in vitro artifact, as no other PNPases are known to possess it, the observation suggested a possible relationship between (p)ppGpp and RNA decay in *Streptomyces*. To begin to examine this relationship, the effects of (p)ppGpp on polymerization and phosphorolysis by *S. coelicolor*, *S. antibioticus*, and *E. coli* PNPases were measured in the absence and presence of (p)ppGpp [59]. As shown in Figure 6, both guanosine penta- and tetraphosphates inhibited the activity of *S. coelicolor* PNPase, in both phosphorolysis and polymerization, though ppGpp was a more potent inhibitor than pppGpp. 

Essentially identical results were obtained for *S. antibioticus* PNPase (not shown). By contrast, neither ppGpp nor pppGpp were effective inhibitors of the activity of *E. coli* PNPase. Indeed, at concentrations up to 1 mM, pppGpp actually stimulated the polymerizing activity of the *E. coli* enzyme.

In the same study, the effects of (p)ppGpp on the stability of bulk mRNA in *S. coelicolor* were examined [59]. It was initially observed that the half-life of bulk mRNA increased by 1.8-fold in stationary phase cultures as compared with exponential phase. That this increase might be related to the effects of (p)ppGpp was suggested by studies with an *S. coelicolor relA* mutant, and a strain containing an inducible *relA* gene. While the half-life of bulk mRNA was longer in the *relA* mutant than in the parental strain (e.g., 8.9 min vs 3.2 min in exponential phase), the half-life decreased slightly in the *relA* mutant in stationary phase (to 7.2 min). In the strain containing an inducible *relA* gene, producing increased levels of (p)ppGpp, induction occasioned a ca. two-fold increase in the half-life of bulk mRNA, from 6.6 to 11.8 min. Taken together, these observations suggest that (p)ppGpp may stabilize mRNAs in stationary phase *S. coelicolor* cells, as compared with cells growing exponentially.

Why and how might this stabilization occur? It is well established that although levels of RNA and protein synthesis decrease dramatically as *Streptomyces* cultures move from the exponential to the stationary phase of growth, a basal level of synthesis is maintained throughout stationary phase [60,61]. This basal level of macromolecular synthesis is presumably required to produce enzymes and other proteins involved in the synthesis of the secondary metabolites these organisms produce in stationary phase. Stabilization of the transcripts for these proteins would represent one strategy the organisms could employ to ensure the persistence of macromolecular synthesis to support secondary metabolite production. It is known that (p)ppGpp is present in significant amounts, even in stationary phase streptomycete cultures [62,63]. Thus, the inhibition of PNPase by (p)ppGpp might represent a strategy used by *Streptomyces* to stabilize essential mRNAs during stationary phase. It would be interesting to determine whether (p)ppGpp inhibits the activity of other exo- and endonucleases and while such analyses have yet to be performed, it is noteworthy that ppGpp inhibits PNPase from another actinomycete, *Nonomuraea* sp. [64].

## 6. Conclusions and Unanswered Questions

It is apparent from the brief analysis above that PNPase is a multitalented enzyme that plays a critical role in the metabolic activities of bacterial cells. Despite the wealth of information that has been accumulated about PNPase, a number of important biological questions remain unanswered, particularly as they relate to the functions of *Streptomyces* PNPase.

First, why is the *rpsO–pnp* operon of *S. coelicolor* transcribed from four promoters? The answer to this question may relate to the fact that the operon contains a ribosomal protein gene, as well as the gene for PNPase. It is possible that the promoters are not only critical to the regulation of *pnp* expression, but that they also play and important role in ribosome biogenesis via their regulation of the levels of the *rpsO* transcript. Mutation of the four promoter sequences may provide insight into these possibilities.

Second, what is the significance, if there is any, to the fact that the *S. coelicolor rpsO–pnp* operon produces six transcripts (two *rpsO* transcripts from P*rpsO*A and B, two readthrough transcripts from the same two promoters, and two *pnp* transcripts from P*pnp*A and B)? It should be noted that Northern blot analysis of the transcripts derived from the *S. coelicolor rpsO–pnp* operon did not reveal the presence of six separate transcripts [65]. It is possible that the different transcripts are not sufficiently different in size to have been resolved on the Northern blotting gels. Another intriguing possibility is that the longer transcripts, obtained from the upstream promoter in each case, might be processed at their 5′-ends by RNase J, which possesses 5′–3′-exoribonuclease activity. RNase J has recently been characterized in *S. coelicolor* [66,67].

Third, as described above, PNPase is a cold shock protein in *Streptomyces*. It is relevant to ask whether PNPase responds to other environmental stresses, such as heat, oxidative stress, metal ion stress, etc. It would be of interest, in particular, to examine the effects of various types of stress on the activities of the *rpsO–pnp* promoters. Mutational analyses again might reveal important aspects of promoter function in stress conditions in *Streptomyces*.

Fourth, a number of small effector molecules modulate the activity of *E. coli* PNPase, e.g., ATP, citrate, and cyclic-diGMP [16,17,18,19]. It has been proposed that these effectors connect RNA decay to other metabolic pathways in bacterial cells. It would be interesting to determine whether these effectors also affect the activity of *Streptomyces* PNPase. It is noteworthy, in this regard, that in silico molecular docking studies suggest that citrate, which inhibits the activity of *E. coli* PNPase, will bind to PNPase from *S. antibioticus* [18].

Finally, in *E. coli* and other organisms, PNPase is part of a larger macromolecular complex generally referred to as the degradosome ([68,69]. In *E. coli*, the components of the degradosome are organized around a scaffold provided by the single strand specific endoribonuclease, RNase E [70]. RNase E is present in *S. coelicolor*, and has been shown to interact with PNPase in vivo [71]. However, unlike the situation in *E. coli*, the identities of other proteins that might be involved in the degradative machine are unknown in *Streptomyces*.

It is fervently hoped that the foregoing and other important questions related to PNPase structure and function will continue to attract interest and experimentation to provide answers to them.
